# Analysis of the MTHFR C677T variant with migraine phenotypes

**DOI:** 10.1186/1756-0500-3-213

**Published:** 2010-07-28

**Authors:** Annie Liu, Saraswathy Menon, Natalie J Colson, Sharon Quinlan, Hannah Cox, Madelyn Peterson, Thomas Tiang, Larisa M Haupt, Rod A Lea, Lyn R Griffiths

**Affiliations:** 1 Genomics Research Centre, School of Medical Science, Griffith University Gold Coast, PMB 50, Gold Coast Mail Centre, Queensland, Australia, 9726; 2 School of Biomolecular & Biomedical Science, Griffith University, Nathan, Queensland, Australia; 3 Institute of Environmental Science and Research, 34 Kenepuru Drive, Porirua Wellington, New Zealand

## Abstract

**Background:**

The methylenetetrahydrofolate reductase (MTHFR) gene variant C677T has been implicated as a genetic risk factor in migraine susceptibility, particularly in Migraine with Aura. Migraine, with and without aura (MA and MO) have many diagnostic characteristics in common. It is postulated that migraine symptomatic characteristics might themselves be influenced by MTHFR. Here we analysed the clinical profile, migraine symptoms, triggers and treatments of 267 migraineurs previously genotyped for the MTHFR C677T variant. The chi-square test was used to analyse all potential relationships between genotype and migraine clinical variables. Regression analyses were performed to assess the association of C677T with all migraine clinical variables after adjusting for gender.

**Findings:**

The homozygous TT genotype was significantly associated with MA (*P *< 0.0001) and unilateral head pain (*P *= 0.002). While the CT genotype was significantly associated with physical activity discomfort (*P *< 0.001) and stress as a migraine trigger (*P *= 0.002). Females with the TT genotype were significantly associated with unilateral head pain (*P *< 0.001) and females with the CT genotype were significantly associated with nausea (*P *< 0.001), osmophobia (*P *= 0.002), and the use of natural remedy for migraine treatment (*P *= 0.003). Conversely, male migraineurs with the TT genotype experienced higher incidences of bilateral head pain (63% vs 34%) and were less likely to use a natural remedy as a migraine treatment compared to female migraineurs (5% vs 20%).

**Conclusions:**

MTHFR genotype is associated with specific clinical variables of migraine including unilateral head pain, physical activity discomfort and stress.

## Background

Migraine is a complex, multifactorial disorder that affects approximately 12% of the Caucasian population [1]. At present, there are no biochemical tests to confirm the diagnosis of migraine; with diagnosis usually achieved by matching the patient's clinical manifestations to the classifications outlined by the International Headache Society (IHS) [2]. The IHS defines two main classes of migraine: migraine with aura (MA) and migraine without aura (MO) [2]. Whilst the two subtypes have significant symptomatic overlap, individuals with MA experience a distinct phase of neurological disturbances known as an "aura", that usually precedes the headache phase of an attack [3,4].

The human MTHFR gene mapped to chromosome 1p36.3 catalyses the nicotinamide adenine dinucleotide phosphate (NADPH) dependent conversion of 5, 10-methylenetetrahydrofolate (CH_2_-THF) to 5-methyltetrahydrofolate (CH_3_-THF), the principal circulatory form of folate and a cofactor for methylation of homocysteine to methionine [5,6]. An increase in circulatory homocysteine levels have been reported in patients with MA [7]. It is proposed that homocysteine acts as an excitatory amino acid in migraine pathophysiology, either by causing vasodilation of cerebral blood vessels or temporary thrombosis of cerebral blood vessels, reducing oxygen into the brain [8,9]. Individuals carrying the C677T variant in the MTHFR gene have been shown to have decreased MTHFR enzyme activity, and the TT genotype is indirectly linked to mild hyperhomocystinemia possibly resulting in vascular disease. The TT genotype has been reported to be a modest, yet significant risk factor for stroke and hypertension [10,11]. The atherothrombotic effects of hyperhomocystinemia have been postulated to increase the risk of stroke and the decrease in MTHFR activity due to C677T mutation, affecting DNA repair and cell division, may result in hypertension [12].

The C677T allele (rs1801133), a common variant of the MTHFR gene has a frequency of approximately 23-41% in the Caucasian population [8,9,13]. Individuals homozygous for this variant express approximately 30% of the mean activity of MTHFR enzyme levels, as compared with individuals without the substitution allele [13,14]. The association of the C677T variant with MA was first reported in a Japanese population and subsequently replicated in both Turkish and Dutch population [8,15,16].

We investigated the MTHFR C677T variant in migraine in an Australian Caucasian population and have similarly shown significant over-representation of the TT genotype in individuals with MA in comparison to the control group [17]. This association was however not seen in a Finnish study that investigated the contribution of the C677T variant in MA and MO patients. Interestingly, Schurks et al. [18] investigated the interrelationships of the MTHFR C677T variant, migraine and cardiovascular disease, with data suggesting a protective effect for the TT genotype against MA in their population [18]. This inconsistency may be a result of allelic heterogeneity, diagnostic variation or differences between the populations examined [19].

As multiple genes have now been associated with migraine susceptibility, it is plausible to assume that different genotypes and susceptibility genes may cause varying disease manifestation [20,21]. Nyholt et al. [21], through genome wide latent-class analysis (LCA) of migraineurs, identified significant linkage on chromosome 5q21 and suggestive linkage on chromosomes 8, 10 and 13 in relation to migraine phenotypes. The study investigated the broad contribution of chromosomal loci to migraine clinical symptoms through linkage analysis, but did not consider the effects of specific genes or polymorphisms within genes [21]. A recent study by Tietjen et al. [22], that investigated if angiotensin converting enzyme (ACE) and MTHFR gene variants are associated with von Willebrand factor (vWF) activity, an endothelial dysfunction marker, and with a distinct headache phenotype in premenopausal women with migraine, observed elevated vWF activity to be associated with the ACE DD genotype, which was highest when combined with the MTHFR TT genotype [22].

The aim of the current study was to investigate migraine phenotypes in relation to the MTHFR gene. This study examined the genotype-phenotype correlations between the C677T variant and the clinical phenotypes of migraine to determine if the MTHFR genotype was associated with migraine in general or more specifically with particular migraine sub-types, symptoms, severity, gender and/or response to medication.

## Methods

Details on the study population, questionnaire administered, phenotypic variables investigated, and the methods used for DNA extraction from blood, polymerase chain reaction, genotyping and statistical analyses are provided in additional file 1.

## Results

### MTHFR genotypes associated with migraine clinical variables

Table 1 shows results for MTHFR genotype analysis in relation to migraine clinical variables. There were 165 MA and 102 MO participants in this study group. Sixteen participants who experienced both MA and MO were classified as having MA as they may share inherited and acquired factors predisposing them to aura. The MTHFR group consisted of 27% males and 73% females. 94% of individuals with the MTHFR TT genotype, suffered from MA as compared to 61% and 55% of individuals carrying the CC and CT genotypes respectively. These results confirm our previous observations [1,17].

**Table 1:** Chi square analysis of MTHFR genotype for individuals experiencing clinical variables versus all migraineurs who do not.

Clinical variables	Pearson's chi-square	N	*P*-value
Migraine Subtype Diagnosis	16.65		< 0.001
Visual disturbances	5.57	96	0.062
Numbness & Tingling	1.49	45	0.475
Speech problems	1.41	26	0.493
Nausea	0.15	165	0.927
Emesis	0.69	121	0.708
Diarrhoea	2.3	18	0.316
Phonophobia	2.16	153	0.34
Photophobia	0.85	177	0.654
osmophobia	3.78	42	0.151
Altered Vision	0.52	81	0.77
Dizziness/Double Vision	4.33	49	0.115
Speech problems	1.78	31	0.411
Numbness & Tingling	0.1	49	0.952
Weakness	2.7	54	0.259
Pulsating & throbbing head pain	1.23	158	0.542
Unilateral head pain	7.07	123	0.029
Bilateral head pain	2.76	66	0.251
Head movement discomfort	2.72	123	0.257
Eye Movement discomfort	3.59	97	0.166
Physical Activity discomfort	8.97	149	0.011
Migraine triggers			
Menses	1.224	58	0.542
Weather Changes	0.98	36	0.613
Stress	7.3	123	0.026
Holiday & Relaxation	3.29	17	0.193
Red Wine	2.5	33	0.286
Other Alcohol	1.48	35	0.478
Chocolate	0.5	46	0.799
Oranges	0.21	23	0.902
Ripe Cheese	4.32	23	0.115
Migraine treatment			
Yes/No	0.04	58	0.981
5-HT1 Drugs	0.03	30	0.985
5-HT1 Drug treatment effectiveness	1.97	21	0.373
Pain killers	5.02	137	0.081
Natural remedy	1.77	27	0.412
Medication for nausea	2.75	31	0.253
Treatment for other conditions/pains			
Yes/No	0.67	17	0.716
Chronic neck pain	1.98	33	0.372
High blood pressure	0.08	16	0.916
Stroke	0.93	1	0.629
Heart Disease	1.5	7	0.473
Depression	1.68	23	0.431
Anxiety disorder	0.32	2	0.853
Panic disorder	0.21	6	0.899
Chronic fatigue	1.71	5	0.425
Irritable bowel	3.22	15	0.2
Menstrual problems	1.19	14	0.551
Chronic back pain	0.95	1	0.623
Contraceptive Pill	0.177	67	0.915
Passive Smoking	0.1	81	0.952

*P values are values of χ^2 ^tests (2tailed) for the 2 × 3 tables (df = 2).

Of the 50 variables tested and after Bonferroni correction for multiple testing, migraine diagnosis (*P *< 0.0001, degree of freedom (df) = 2), unilateral head pain (*P *= 0.002, df = 2), physical activity discomforts (*P *< 0.001, df = 2) and stress as a migraine trigger (*P *= 0.002, df = 2) were all factors that were significantly associated with MTHFR genotype (Table 2). Further analyses demonstrated the TT genotype to be significantly associated with MA (P < 0.0001) and unilateral head pain (P = 0.002). Frequency data found 87% of migraineurs with the TT genotype experienced unilateral head pain compared to 62% in the CC group. The CT group showed an intermediate percentage of migraineurs who experienced unilateral head pain (77%). A smaller percentage of migraineurs carrying the CC genotype experienced discomfort associated with physical activity during or just prior to migraine (69%), while the TT group had an intermediate response (83%) and the CT (88%) group had the highest response (P < 0.001). The CT genotype group also had the highest percentage of participants acknowledging stress as a migraine trigger (76%) (P = 0.002) (Table 2). These findings suggest that individuals carrying one and/or more copies of the T allele are more prone to unilateral head pain, are more likely to experience discomfort associated with physical activity during or prior to migraine and have stress as a migraine trigger compared to those carrying the CC genotype.

**Table 2:** Genotypic frequency distribution of MTHFR and statistically significant clinical variables in relation to MTHFR genotype

Clinical variables	Subtype (Total)	MTHFR Genotype %		
		CC	CT	TT
Migraine Diagnosis	MO (102)	39 (39%)	61 (45%)	2 (6%)
*P *< 0.0001	MA (165)	61 (61%)	74 (55%)	30 (94%)
Unilateral head pain	Y (123)	40 (62%)	63 (77%)	20 (87%)
*P *< 0.0001	N (47)	25 (38%)	19 (23%)	3 (13%)
Physical activity	Y (149)	51 (69%)	78 (88%)	20 (83%)
*P *< 0.001	N (38)	23 (31%)	11 (12%)	4 (17%)
Stress	Y (123)	42 (61%)	68 (76%)	13 (52%)
*P *= 0.002	N (60)	27 (39%)	21 (24%)	12 (48%)

Significant *P *values after correction for multiple testing (Bonferroni correction).

MO = Migraine without aura, MA = Migraine with aura, Y = Yes and N = No.

To further analyse the contribution of the recessive TT genotype with migraine clinical variables, the CC and the CT groups were reclassified into one group, and compared with the TT genotype group. The chi-square, Kruskal-Wallis and Kolmogorov-Smirnov Z tests between the two reclassified genotype groups and migraine clinical data revealed statistical significance of genotype with migraine diagnosis (*P *< 0.000, df = 1) and visual disturbances *(P*= 0.001, df = 1). Frequency data demonstrated the recessive TT genotype group had a significantly higher percentage of individuals with MA; while the CC/CT genotype group appeared to have an equal distribution of the two different migraine subtypes.

### Gender differences and the MTHFR genotype

The inclusion of gender as an independent variable revealed gender specific statistical significance for a number of migraine clinical variables. In female migraineurs the TT genotype was significantly associated with unilateral head pain (OR = 0.35, CI-95 = 0.15-0.79, *P *< 0.001). Conversely, male migraineurs experienced higher incidences of bilateral head pain, especially in those with the TT genotype, compared to female migraineurs (67% vs 19%)(Table 3). In addition, female migraineurs with the CT genotype were more likely to suffer from nausea (OR = 3.41, CI-95 = 1.44-8.08, *P *< 0.001), osmophobia (OR = 3.39, CI-95 = 1.01-10.48, *P *= 0.002) and use natural remedy as a migraine treatment (OR = 0.2, CI-95 = 0.05-0.92, *P *= 0.003) compared to male migraineurs (Tables 4).

**Table 3:** Genotypic frequency distribution of MTHFR for statistically significant clinical variables in relation with gender

Clinical variables	Gender	MTHFR Genotype %		
		CC	CT	TT
Nausea	M	10(100%)	15(60%)	5(71%)
	F	55(85%)	64(94%)	16(89%)
Osmophobia	M	1(13%)	3(14%)	0(0%)
	F	15(26%)	21(35%)	2(13%)
Unilateral head pain	M	4(50%)	15(65%)	3(50%)
	F	36(63%)	48(81%)	17(100%)
Bilateral head pain	M	5(62%)	13(62%)	4(67%)
	F	26(46%)	15(26%)	3(19%)
Natural Remedy	M	0(0%)	2(9%)	0(0%)
	F	8(15%)	14(25%)	3(19%)

**Table 4:** Statistically significant clinical variables by gender and MTHFR genotype

Clinical variables	*P*-value	OR	OR (95% CI)
Nausea	< 0.001	3.41	(1.44-8.08)
Osmophobia	= 0.002	3.39	(1.01-10.48)
Unilateral head pain	< 0.001	0.35	(0.15-0.79)
Bilateral head pain	< 0.000	0.25	(0.11-0.55)
Natural remedy	= 0.003	0.2	(0.05-0.92)

Significant *P *values after correction for multiple testing (Bonferroni correction).

## Discussion

Migraine is most likely produced as a result of the interaction between multiple genes with environmental factors and triggers. As a consequence, variability and overlap is expected in the manifestations of the associated genetic defect/s. In a study of the clinical manifestations associated with mutations in the calcium- channel, voltage dependent, P/Q type, alpha A subunit gene (CACNA1A) in 28 families with Familial Hemiplegic Migraine type 1, Ducros et al (2001) [3], revealed significant genotype-phenotype correlations. In addition to clinical variability being partly due to the different CACNA1A mutations, the study also suggested that variability in phenotypic expression among patients with the same mutation could be influenced by other genetic or environmental factors.

The study of vascular genes in migraine identified a role for MTHFR gene. MTHFR synthesizes 5-methylenetetrhydrofolate, the major carbon donor required for efficient remethylation of homocystein to methionine [23]. The MTHFR C677T allele results in an amino acid change and reduces MTHFR enzyme activity leading to mild hyperhomocysteninemia [13]. Hyperhomocysteninemia have been suggested to produce endothelial cell injury in animal and cell culture studies; this homocysteine related dysfunction of the vascular endothelium may potentially influence migraine susceptibility, especially MA, through the activation of trigeminal fibres [24-26]. It is possible that the vascular disturbance connected to hyperhomocysteinemia trigger downstream neurological manifestations that are observed in MA sufferers.

The current study investigated genotype-phenotype correlations of the migraine susceptibility gene, MTHFR with 50 migraine clinical variables. Even after correction for multiple testing, analyses indicated the MTHFR genotype to be significantly associated with migraine diagnosis, unilateral head pain, physical activity discomforts, and stress as a migraine trigger. The homozygous MTHFR TT genotype was linked with MA and unilateral head pain and the heterozygous CT genotype was linked to physical activity discomforts during or prior to migraine and stress as a migraine trigger. While the TT and CC genotypes showed the highest and the lowest percentage of participants suffering from unilateral head pain during or prior to a migraine respectively, the CT genotype clearly demonstrated an intermediate response. Frequency data of participants suffering from physical activity discomfort during or prior to migraine showed the CT and the CC genotypes to have the highest and the lowest percentage of participants respectively and the TT genotype showed an intermediate response. Interestingly, the CT and the TT genotypes showed the highest and the lowest number respectively, of migraineurs reporting stress as a migraine trigger.

When the MTHFR genotype groups were examined by comparing CC and CT genotypes to the homozygous TT genotype, the resulting regression models did not alter drastically. The TT genotype remained significantly linked to MA and was also observed to be significantly associated with visual disturbance. When gender differences were investigated in relation to genotype and phenotype it was found that bilateral head pain was observed more commonly in male migraineurs. In contrast, in females, nausea, unilateral head pain, osmophobia and the use of natural remedy as a migraine treatment were significantly associated with one or more copies of the T allele. The gender distribution in this study is not equal with 27% of the participants being males and 73% of the participants being females; some caution has to be exercised when interpreting the significant results. Gender differences in relation to genotype and phenotype have to be examined in a bigger cohort to look at the effect of MTHFRC677T genotype on migraine clinical symptoms diligently.

The TT genotype of the MTHFR C677T variant has been shown in several studies including a recent meta-analysis by Rubino et al [27] to confer a modest risk for MA [27]. This study expanded on this association to examine genotype-phenotype correlations between the C677T variant and the clinical phenotypes of migraine. The presence of the T allele was associated with the largest number of migraine symptoms and triggers, suggesting that although the TT genotype appears to have a recessive effect on MTHFR enzyme levels, perhaps both heterozygous and homozygous states of the T allele may contribute to the phenotypic expression of migraine. As well as intrinsic enzyme levels, individuals with the CT genotype may be more susceptible to the environmental triggers associated with migraine attacks.

The effects of elevated levels of homocysteine on neurons have been reported to include DNA damage, altered DNA repair, disturbance in DNA methylation and oxidative stress [26,28-30]. Animal studies have reported cytotoxic effects of high levels of homocysteine to included apoptosis in sensitive brain areas such as the striatum and cerebellum involved in motor function and altered neurobehavioural capacity in rat models [31]. It is thus plausible that the resulting levels of homocysteine conferred by the T allele may contribute to selected phenotypic expressions described in migraineurs.

The major limitation of the present study was the number of available subjects in comparison to the number of outcome variables examined. As such, further investigations utilising larger populations to clarify the genotype-phenotype interactions of the migraine susceptibility gene MTHFR are warranted.

## Conclusion

This study examined the contribution of the C677T genotype of the migraine susceptibility gene MTHFR, to migraine subtypes, triggers, severity, symptoms and response to medication. Interestingly, the TT genotype of the MTHFR gene, while being significantly correlated to MA as expected, was also found to be associated with the largest number of migraine symptoms and triggers.

## Competing interests

The authors declare that they have no competing interests.

## Authors' contributions

AL performed experimental procedures, participant sample preparation and contributed towards the statistical analysis of the study. SM performed experimental procedures, participant sample preparation and contributed towards the statistical analysis and manuscript finalisation. SQ and MP contributed towards participant recruitment and data management. NJC, HC, TT and LMH contributed towards data interpretation and manuscript finalisation. RAL contributed towards data interpretation, statistical analysis and manuscript finalisation. LRG participated in the conception and design of the stud, data analysis and interpretation and coordinated the study. All authors have reviewed and approved the manuscript.

## Supplementary Material

Additional file 1 **Detailed Methods**. A detailed description of the methods used in recruitment and selection of participants used for this study, including genotyping and statistical analyses. Click here for file
