# Supplementary material for: Analysis of the MTHFR C677T variant with migraine phenotypes
Source: BMC Res Notes. 2010 Jul 28;3:213. doi: 10.1186/1756-0500-3-213 (PMC2919563; doi:10.1186/1756-0500-3-213)
Supplement: Additional file 1 — Detailed Methods. A detailed description of the methods used in recruitment and selection of participants used for this study, including genotyping and statistical analyses. [file 1756-0500-3-213-S1.DOC]

# Methods

## Study subjects

Study participants ( n = 267) were drawn from two large age (+/- 5 years) and sex matched case-control panels previously genotyped for the MTHFR C677T mutation [1]. All participants were Caucasians with United Kingdom descent recruited from the East Coast of Australia. Participants were interviewed and asked to complete a detailed questionnaire that was administered through Griffith University’s Genomic Research Centre (GRC), providing information including personal and family medical history, migraine symptoms, age of onset, frequency, severity and migraine treatment as previously described [2, 3].Those suffering from migraine were diagnosed as having MA or MO through interview by an experienced neurologist in combination with their answers to the questionnaire that was prepared using the International Headache Society (IHS) criteria (HCCIHS 2004). There were 165 MA and 102 MO participants in this study. Participants who experienced both subtypes of migraine were classed as being affected with MA. Individuals reported affected with, or had a family history of known migraine or comorbid conditions such as mental illness (including depression and schizophrenia) cerebral vascular disease and alcohol, were excluded from the study. Written informed consent was obtained from all participants and the study was approved by the Griffith University Ethics Committee for Experimentation on Human Subjects.

## Phenotype variables

The dependent (outcome) variables for the study were migraine clinical data related information of which there were four main categories. These were: i) migraine subtype diagnosis, ii) migraine triggers, iii) migraine treatment and iv) treatment for other conditions/pains. In total, there were around 50 outcome variables under these main categories, a summary of which are outlined in Table 1. Each of these dependent variables underwent statistical analysis against genotype. The independent MTHFR variable has three possible genotypes, CC, CT and TT.

## Genotyping

Genomic DNA was isolated from whole blood by standard salting out method as previously described by Miller et al [4]. DNA fragments with the MTHFR C677T variant were PCR amplified and the resulting product was digested by the H*inf*  1 enzyme and fractionated using a 5% ultra –high-resolution agarose gel. The genotype results were confirmed using an ABI-3130 genetic analyser. The detailed genotyping method for the samples used in this study has been previously published in Lea et al 2004 [1].

## Statistical Analysis

Statistical analysis of the variables was performed using SPSS for windows version 13. The chi-square statistical test was used to test for potential relationships between genotype and all migraine qualitative dependent variables as well as the relationship between migraine diagnosis and migraine dependent variables. For quantitative outcome variables, nonparametric tests of variance, the Kruskal-Wallis, Median, Mann-Whitney and Kolmogorov-Smirnov tests were used. Logistic and ordinal regression analyses were used to devise models where more than one independent variable (ie. genotype and gender) was used to predict the outcome of dependent variables. Regression analyses were performed to identify whether the addition of gender improved the model compared to simply predicting with genotypes alone. The equivalent number of measured independent traits was measured using the matSpD interface (http://genepi.qimr.edu.au/general/daleN/matSpD)[5, 6]. matSpD analysis determined that the original 50 variables correspond to approximately 14 independent traits. Bonferroni correction was applied to correct for multiple testing and to determine the significance of the results by dividing the significance level by the number of independent traits. The level of significance was taken at P value of .05/14 = 0.004[7].

## References

**1. Lea, R.A., et al., *The methylenetetrahydrofolate reductase gene variant C677T influences susceptibility to migraine with aura.* BMC Med, 2004. 12(2): p. 3**

**2. Colson, N.J., et al., *The estrogen receptor 1 G594A polymorphism is associated with migraine susceptibility in two independent case/control groups.* Neurogenetics, 2004. 5(2): p. 129-33.**

**3. Lea, R.A., et al., *Genetic variants of angiotensin converting enzyme and methylenetetrahydrofolate reductase may act in combination to increase migraine susceptibility.* Brain Res Mol Brain Res, 2005. 136(1-2): p. 112-7.**

**4. Miller, S.A., D.D. Dykes, and H.F. Polesky, *A simple salting out procedure for extracting DNA from human nucleated cells.* Nucleic Acids Res, 1988. 16(3): p. 1215.**

**5. Li, J. and L. Ji, *Adjusting multiple testing in multilocus analyses using the eigenvalues of a correlation matrix.* Heredity, 2005. 95(3): p. 221-7.**

**6. Nyholt, D.R., *A simple correction for multiple testing for single-nucleotide polymorphisms in linkage disequilibrium with each other.* Am J Hum Genet, 2004. 74(4): p. 765-9.**

**7. Distel, M.A., et al., *Personality, health and lifestyle in a questionnaire family study: a comparison between highly cooperative and less cooperative families.* Twin Res Hum Genet, 2007. 10(2): p. 348-53.**
